# Empowering legal justice with AI: A reinforcement learning SAC-VAE framework for advanced legal text summarization

**DOI:** 10.1371/journal.pone.0312623

**Published:** 2024-10-25

**Authors:** Xukang Wang, Ying Cheng Wu

**Affiliations:** 1 Sage IT Consulting Group, Shanghai, China; 2 School of Law, University of Washington, Seattle, WA, United States of America; Alexandria University Faculty of Nursing, EGYPT

## Abstract

Automated summarization of legal texts poses a significant challenge due to the complex and specialized nature of legal documentation. Despite the recent progress in reinforcement learning for natural language text summarization, its application in the legal domain has been less effective. This paper introduces SAC-VAE, a novel reinforcement learning framework specifically designed for legal text summarization. We leverage a Variational Autoencoder (VAE) to condense the high-dimensional state space into a more manageable lower-dimensional feature space. These compressed features are subsequently utilized by the Soft Actor-Critic (SAC) algorithm for policy learning, facilitating the automated generation of summaries from legal texts. Through comprehensive experimentation, we have empirically demonstrated the effectiveness and superior performance of the SAC-VAE framework in legal text summarization.

## 1 Introduction

The rapid proliferation of legal documents in the legal system poses a significant challenge for legal professionals and the public alike, necessitating efficient mechanisms for managing and understanding this vast information. Legal summarization is rapidly emerging as a pivotal area of inquiry at the intersection of Machine Learning (ML) and legal studies [1, 2]. Fueled by the burgeoning complexity and volume of legal documents, which encompass contracts, case law, and statutes, there is an escalating imperative for the development of automated legal summarization algorithms [3, 4]. The primary aim of this specialized domain is to distill expansive legal corpora into concise, readily interpretable summaries, thereby streamlining tasks such as predictive modeling of legal judgments without necessitating manual review of the source documents [5]. These automated summaries serve a dual purpose: they not only accelerate the extraction of salient legal information but also facilitate nuanced, comparative analyses across a spectrum of legal jurisdictions. Despite its burgeoning importance, this field is confronted with substantial challenges, chief among them the intricate and dynamically evolving nature of legislation.

In the face of intricate legal documents, traditional summarization approaches typically resort to domain-specific handcrafted features and rudimentary statistical measures tailored for particular classes of legal judgments [6, 7]. With the advent of deep learning techniques and the increasing availability of public legal documents, several initiatives have emerged to develop automated, end-to-end systems for legal text summarization [1]. Prior works in supervised learning for legal text summarization have predominantly employed differentiable loss functions such as cross-entropy, aiming to maximize the likelihood of generating accurate summaries. These approaches have demonstrated superior performance over traditional methods on certain legal datasets [8, 9]. However, they come with the caveat of requiring substantial amounts of labeled data and extended training periods, which is particularly burdensome in the context of legal text summarization.

To tackle this challenge, existing research has turned to reinforcement learning techniques [10, 11], wherein an agent learns to autonomously generate summaries of legal texts through a cycle of trial-and-error interactions with a designated environment. Such reinforcement learning methods offer the advantage of enhancing the quality of document summarization. The summarized content, in turn, serves as a concise yet comprehensive guide to understanding the crux of legal cases [12]. Nevertheless, the deployment of reinforcement learning algorithms in the field of legal text summarization presents significant challenges, largely attributable to their intrinsic trial-and-error learning mechanisms. These encompass complex state spaces, long training cycles, and issues related to model convergence. These obstacles highlight the urgent necessity for the advancement of more efficient methodologies within the specialized domain.

This paper introduces the SAC-VAE framework, a novel combination of RL and VAE, specifically designed to address the unique challenges posed by legal text summarization. By leveraging VAE to reduce high-dimensional state spaces into a lower-dimensional, more manageable feature space, and coupling this with the SAC algorithm for effective policy learning, the SAC-VAE framework offers a more computationally efficient solution. This work is particularly beneficial for legal professionals who require timely, accurate, and concise summaries to handle the ever-growing volume of legal documents. The primary aim of this study is to develop and validate the SAC-VAE framework for legal text summarization. Measurable outcomes include the framework’s performance as demonstrated by ROUGE and BLEU scores, as well as its efficiency in terms of training time and convergence rates when compared to baseline methods.

We firstly validate our SAC-VAE algorithm on the public legal datasets with high-dimensional state spaces. The experimental results on the U.S. legal datasets demonstrate that our model achieves comparable results to state-of-the-art RL methods while the training time required is also drastically reduced. Additionally, we employed the reconstruction error—measured between the vector reconstructed from low-dimensional features and the original high-dimensional state space—as well as visualization results to ascertain the optimal dimensionality for the reduced feature space.

The primary contributions of this paper are threefold:

First, introduction of SAC-VAE, a novel fast deep reinforcement learning framework: This framework utilizes low-dimensional feature extraction on the original state space of deep reinforcement learning, leveraging these compact features to efficiently generate legal summaries.

Second, dimensionality selection for low-dimensional features: A method is proposed for determining the optimal dimensionality of the reduced feature space. This method takes into account both feature reconstruction error and visualization results to arrive at an appropriate low-dimensional feature dimension.

Third, empirical validation: The proposed framework and dimensionality selection method were rigorously evaluated in the context of legal text summarization, thereby substantiating the efficacy of SAC-VAE and the soundness of the chosen low-dimensional feature dimension.

This paper thus offers a comprehensive approach to addressing the complexities of legal text summarization through innovative algorithmic and methodological advancements.

## 2 Related work

The task of summarizing legal documents has garnered increasing attention in recent years, with various techniques being proposed to tackle the unique challenges posed by the complexity and structure of legal texts [1]. While extractive methods are prevalent, they often fall short in capturing the nuanced language and intricate structure of legal documents. Abstractive methods, on the other hand, face challenges in maintaining the accuracy of the generated summaries, especially given the length and complexity of legal documents.

Machine learning approaches, both supervised and unsupervised, have also been explored but are limited by the availability of large labeled datasets, which are often not feasible in the legal domain [13, 14]. Hierarchical models that take into account the document structure offer a more nuanced approach but are computationally expensive and may not scale well for extensive legal documents [15, 16]. Despite the advancements in these techniques, there is a noticeable gap in the literature concerning the application of reinforcement learning methods for legal document summarization.

Diverging from existing literature, our study presents an innovative methodology that capitalizes on the strengths of VAE [17] for dimensionality reduction in the inherently high-dimensional state spaces of legal documents. This compressed feature set is then integrated into the SAC algorithm [18], a model-free reinforcement learning approach, to produce succinct yet comprehensive summaries. Our hybrid framework aims to address the shortcomings of extant methods by synergistically leveraging unsupervised learning for feature extraction and reinforcement learning for decision-making. This approach seeks to provide a more efficient, accurate, and scalable avenue for the summarization of legal documents, thereby minimizing the need for labor-intensive feature engineering and domain-specific acumen. Through the fusion of VAE and SAC technologies, we aspire to chart new territories in the field of legal document summarization, thereby enhancing its precision and accessibility for both legal practitioners and the broader public.

## 3 Preliminaries

### 3.1 Problem formulation

In this section, we formulate the task of legal document summarization as a Markov Decision Process (MDP). For a given legal document *T* comprising *n* sentences *T* = {*s*_1_,*s*_2_,…,*s*_*n*_}, the reinforcement learning (RL) model aims to extract *m* salient sentences (where *m*<*n*), and rearrange them to construct a summary *S*. This task can be interpreted as a binary classification problem, wherein the model assigns a binary label *y*_*i*_∈{0,1} to each sentence. A label of *y*_*i*_ = 1 indicates that the *i*-th sentence is selected for inclusion in the summary. The RL model is trained to allocate a score *π*(*y*_*i*_|*s*_*i*_,*T*,*θ*) to each sentence, quantifying its relevance. Here, *θ* denotes the learned parameters of the policy network. Upon training completion, the model selects the top *m* sentences with the highest scores in *π*(*y*_*i*_|*s*_*i*_,*T*,*θ*) to compose the summary.

### 3.2 Reinforcement learning for legal summarization

To train summarization models using reinforcement learning (RL), existing literature predominantly employs straightforward policy gradient methods for optimization. In this section, we provide an overview of these RL techniques.

The RL objective is the expected total reward over time $J(\theta )=E_{\tau ∼p(\tau )}[\sum_{i=1}^{n}r_{i}(s_{i},y_{i})]$, where *τ* is the sampled trajectory, *s*_*i*_ is the state of the agent, and *y*_*i*_ is the action taken by the agent at the time step *i*, the main idea of *J*(*θ*) is to reinforce good actions to push up the probabilities of actions that lead to a higher total reward, and push down the probabilities of actions that lead to a lower total reward, until the model obtains an optimal policy.

The objective function in reinforcement learning (RL), denoted as *J*(*θ*), is defined as the expected cumulative reward over time: $E_{\tau ∼p(\tau )}[\sum_{i=1}^{n}r_{i}(s_{i},y_{i})]$, where *τ* represents a sampled trajectory, *s*_*i*_ is the state of the agent at timestep *i*. The primary aim of *J*(*θ*) is to amplify the probabilities of actions that yield higher cumulative rewards, while diminishing those that result in lower rewards, until an optimal policy is attained.

In reinforcement learning, the gradient update is highly sensitive to the choice of learning rate. A large learning rate can induce substantial shifts in the policy, potentially destabilizing the learning process. Conversely, an overly conservative learning rate can severely impede the rate of convergence, leading to sluggish learning progress. Such sensitivities are particularly impactful in the context of text summarization: summaries generated under a suboptimal policy can misguide the learning process, increasingly deviating the policy from an optimal solution.

## 4 Methodology

In the context of legal document summarization, the original high-dimensional state space often contains significant redundancy, posing challenges for direct policy learning. To address this, the present study introduces the SAC-VAE architecture, which employs a self-supervised model to distill salient features from the high-dimensional state space. These features serve as inputs to a reinforcement learning framework, thereby enhancing learning efficiency. This section first provides an overview of the SAC-VAE architecture before delving into a detailed exposition of each constituent module.

### 4.1 Algorithm overview

**Fig 1:**
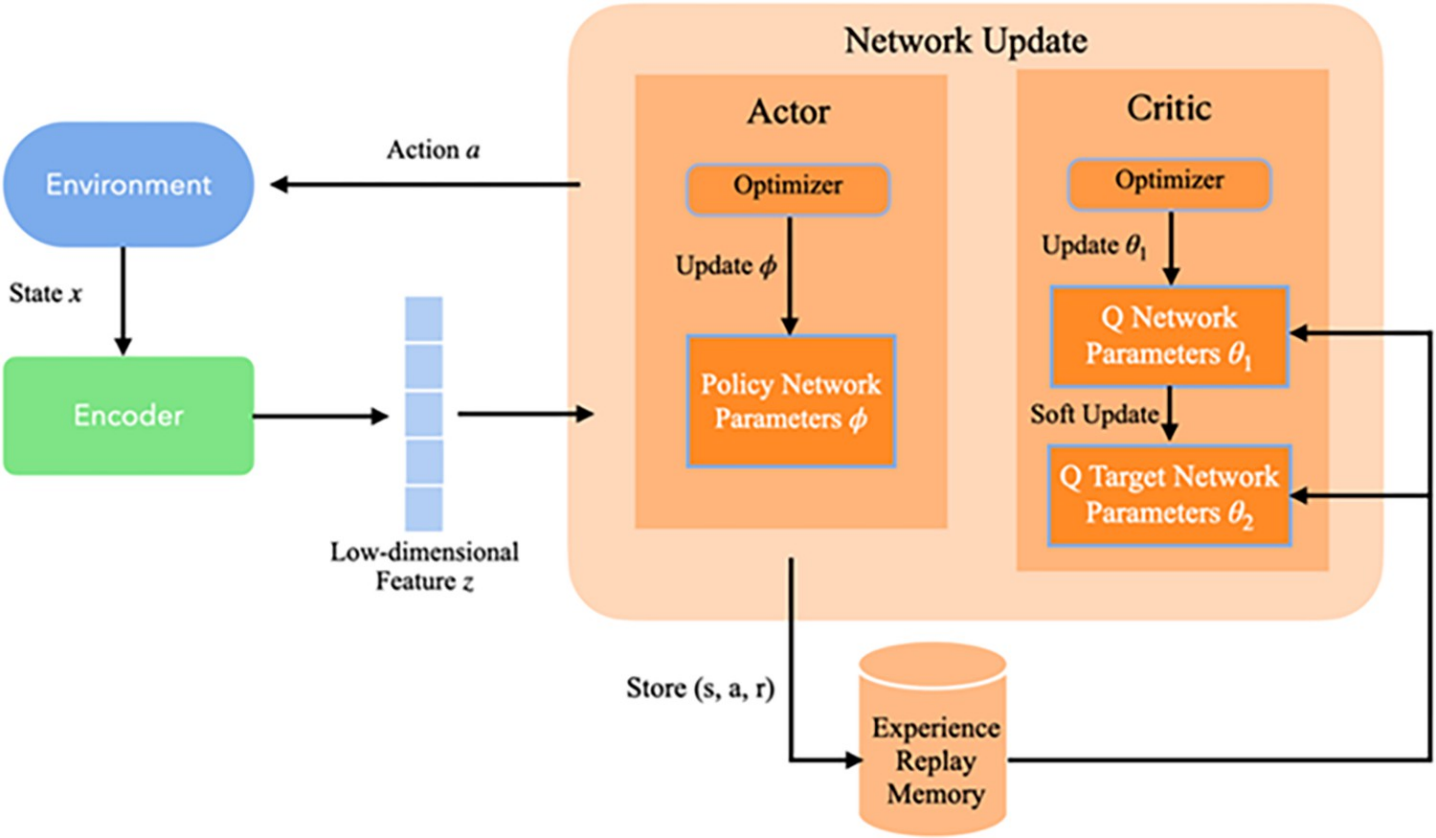
Architecture of SAC-VAE algorithm.

The SAC-VAE framework presented in this article comprises two primary modules: an unsupervised representation learning module and a policy learning module. The former is designed to transform the original high-dimensional state into a compressed, low-dimensional feature. This compressed feature set is subsequently integrated into the policy learning module to facilitate efficient policy optimization. Fig 1 provides a schematic overview of the SAC-VAE algorithm’s architecture.

As depicted in Fig 1, the primary component of the SAC-VAE framework is the policy learning module, which is built upon the SAC framework. This module encompasses a policy network and action value networks. Its objective is to adapt to the reward feedback and state transitions specific to the environment of the legal text summarization task. The module is trained on reconstructed low-dimensional states to derive an action policy. Complementing this is an unsupervised representation learning module for low-dimensional feature generation, implemented as an encoder network using the architecture. This encoder maps the original, high-dimensional state space associated with the legal text summarization task onto a compressed feature space, thereby facilitating accelerated training of the primary policy. Subsequent sections provide a detailed elaboration of each component.

### 4.2 Unsupervised representation learning module

**Fig 2:**
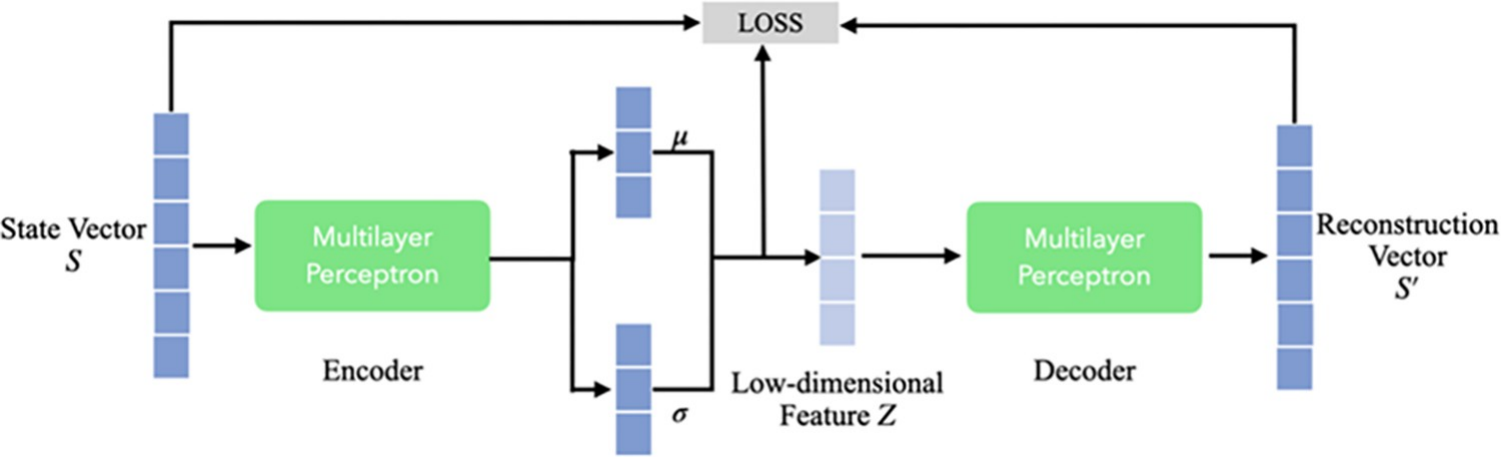
Architecture of unsupervised representation learning module.

The primary objective of the unsupervised feature representation learning module is to distill the original high-dimensional state information into compact, low-dimensional features, while minimizing the loss of essential information. In the absence of supervised data, we employ a VAE to generate these low-dimensional state features [17]. VAE is an unsupervised generative model grounded in variational inference and comprises two main components: an encoder and a decoder. The encoder is tasked with mapping the original high-dimensional feature space onto a low-dimensional space. Specifically, given a state vector *S* defined by the legal text summarization task, the encoder generates an implicit feature vector that follows a Gaussian distribution parameterized by *μ* and *σ*, both of which are produced by the encoder. The decoder, on the other hand, aims to reconstruct the original features, transforming *Z* back to *S*′. This architecture is further illustrated in Fig 2.

In accordance with Bayesian theory, the joint probability distribution of a given state vector *S* and the latent variable *Z* is articulated in Eq 1.


p(z|s)=p(s|z)p(z)/p(s) (1)

However, obtaining *p*(*s*) is computationally challenging, necessitating the introduction of an alternative distribution to approximate *p*(*z*|*s*), This approximate distribution is denoted as *q*_*ω*_(*z*|*s*), representing the posterior model approximated by the encoder. Analogous to the generative model (decoder) *p*_*ψ*_(*s*|*z*)*p*_*ψ*_(*z*), the training process for both the encoder and decoder involves the concurrent optimization of parameters *ω* and *ψ*. This paper jointly trains the approximate posterior model and the generative model by maximizing the variational lower bound, as shown below.


$$\zeta =-D_{KL}(q_{\omega }(z|s)||(p_{\psi }\left(z\right))+E_{q_{\omega }(zs^{\left(i\right)})}[logp_{\psi }(s^{\left(i\right)}z)]$$ (2)

Assuming that *p*_*ψ*_(*z*) follows a normal distribution, as defined in Eq 3, *z* is obtained by Gaussian sampling from Eq 4.


$$p_{\psi }(z)∼N(0,1)$$ (3)

$$q_{\omega }(z|s^{\left(i\right)})∼N(\mu^{\left(i\right)},\sigma^{2(i)})$$ (4)

Therefore, the loss function of this model includes two parts: KL divergence and reconstruction loss, and the derivation results are shown in Eq 5.


$$\zeta =\frac{1}{2n}\sum_{j=1}^{n}\{\lambda \sum_{j=1}^{n}\mu_{j}^{2}+\sigma_{j}^{2}-1-log\;\sigma_{j}^{2}\}+\frac{1}{2n}\sum_{i=1}^{n}||s_{i}-s_{i}^{\prime }|{|}^{2}$$ (5)

From the above formula, *D*_*KL*_(*q*_*ω*_(*z*|*s*))||*p*_*ψ*_(*z*)) represents the approximation ability of the approximate posterior model. $E_{q_{\omega }(z|s^{\left(i\right)})}[logp_{\psi }\left(s^{\left(i\right)}\right|z)]$represents the ability of the generative model to reconstruct *s*′ based on *z*. Consequently, this methodology enables us to generate low-dimensional features from the original state space associated with legal text summarization. In doing so, we acquire a reconstructed state that closely approximates the original state information.

### 4.3 Policy learning module

Leveraging the low-dimensional feature generation module, we can produce a low-dimensional feature vector corresponding to the environment’s original state, thereby facilitating subsequent policy learning. To optimize the efficiency of policy training, this section employs the SAC framework as the principal architecture for policy learning. Grounded in entropy maximization theory, this framework ensures a balanced trade-off between maximizing expected returns and entropy during network updates. This approach enhances the network’s exploratory capabilities and expedites the learning process. The corresponding objective function is delineated in Eq 6.


$$\pi^{*}=argmax_{\pi }E_{s_{t},a_{t}∼\pi }[\sum_{t=0}^{\infty }\gamma^{t}r(s_{t},a_{t})+\alpha H(\pi (∙|S_{t}))]$$ (6)

$$H(\pi (∙|S_{t})=E[-log\pi (∙|S_{t})]$$ (7)

In Eq 6, the formula serves to update the policy *π* that maximizes the total reward. Here, *α* represents the entropy regularization coefficient, employed to modulate the significance of entropy in the optimization process. Eq 7 defines the entropy value, with a larger entropy value correlating to a higher degree of exploration by the agent.

**Table:** 

Algorithm 1 Unsupervised representation learning module	
1	Initialize: Đ, *q*_*ω*_(*z*|*s*), *p*_*ψ*_(*s*|*z*), *ω*, *ψ*
2	while (*ω*, *ψ*) not convergence do
3	ℳ ~ Đ
4	z = random samples from Gaussian distribution(μ,σ2)
5	compute *ζ* and its gradients update (*ω*, *ψ*)
6	End
7	return *ω*, *ψ*

The learning process is delineated in the pseudocode of the following algorithm.

**Table:** 

Algorithm 2 SAC-VAE framework	
1	Initialize: Nencoder in VAE, Initialize *θ*_1_, *θ*_2_, *φ* for q-network and policy network arbitrarily.
2	$\bar{\theta_{1}}=\theta_{1}$, $\bar{\theta_{2}}=\theta_{2}$, Initialize experience replay memory Đ
3	for each iteration do
4	for each environment step do at = πφ(at|st) st+1 = p(st+1|st, at) s’t = Nenc(st) s’t+1 = Nenc(st+1)
5	
6	
7	
8	
9	Đ = Đ∪ {s’t,at,rt, s’t+1}
10	end for
	for each gradient step do
11	Sample from D;
12	The parameters *θ*_1_ and *θ*_2_ of the action value network, along with the policy network parameter *φ*, are updated in accordance with Eqs 6 and 7.
13	Update the entropy regularization coefficient *α*
14	end for
15	end for

## 5 Experiment

To substantiate the efficacy of the proposed method, this section undertakes a comprehensive experimental evaluation centered on the task of legal text summarization. The experiments are designed to address three primary objectives: (1) a comparative assessment between the approach presented in this paper and established baseline algorithms, using a consistent legal text dataset; (2) an exploration of optimal low-dimensional feature dimensions, with performance analyses of the algorithm under varying degrees of feature compression; and (3) a similarity analysis between the low-dimensional reconstructed state features and their original high-dimensional counterparts, aimed at evaluating the algorithm’s reconstruction fidelity.

### 5.1 Dataset

This study employs the BillSum dataset, which comprises 22,218 U.S. Congressional bills accompanied by human-generated reference summaries, sourced from the United States Government Publishing Office [6]. The dataset is partitioned into 18,949 training instances and 3,269 test instances. On average, each document in the dataset contains approximately 46 sentences, while the corresponding summary typically consists of around six sentences.

In this study, the baseline algorithm employed for comparison utilizes the SAC framework [18], an approach grounded in entropy maximization theory. This ensures that during network updates, a balance is struck between maximizing expected returns and entropy, thereby enhancing exploratory capabilities and expediting the learning process. This algorithm exhibits good performance in the context of legal text summarization tasks. The proposed SAC-VAE framework was validated using the BillSum dataset, a widely recognized and validated dataset in the field of legal text summarization. We further ensured the robustness of our results by testing the model with multiple random seeds and evaluating its performance under various conditions to ensure replicability and reliability.

### 5.2 Comparison with baseline algorithm

**Fig 3:**
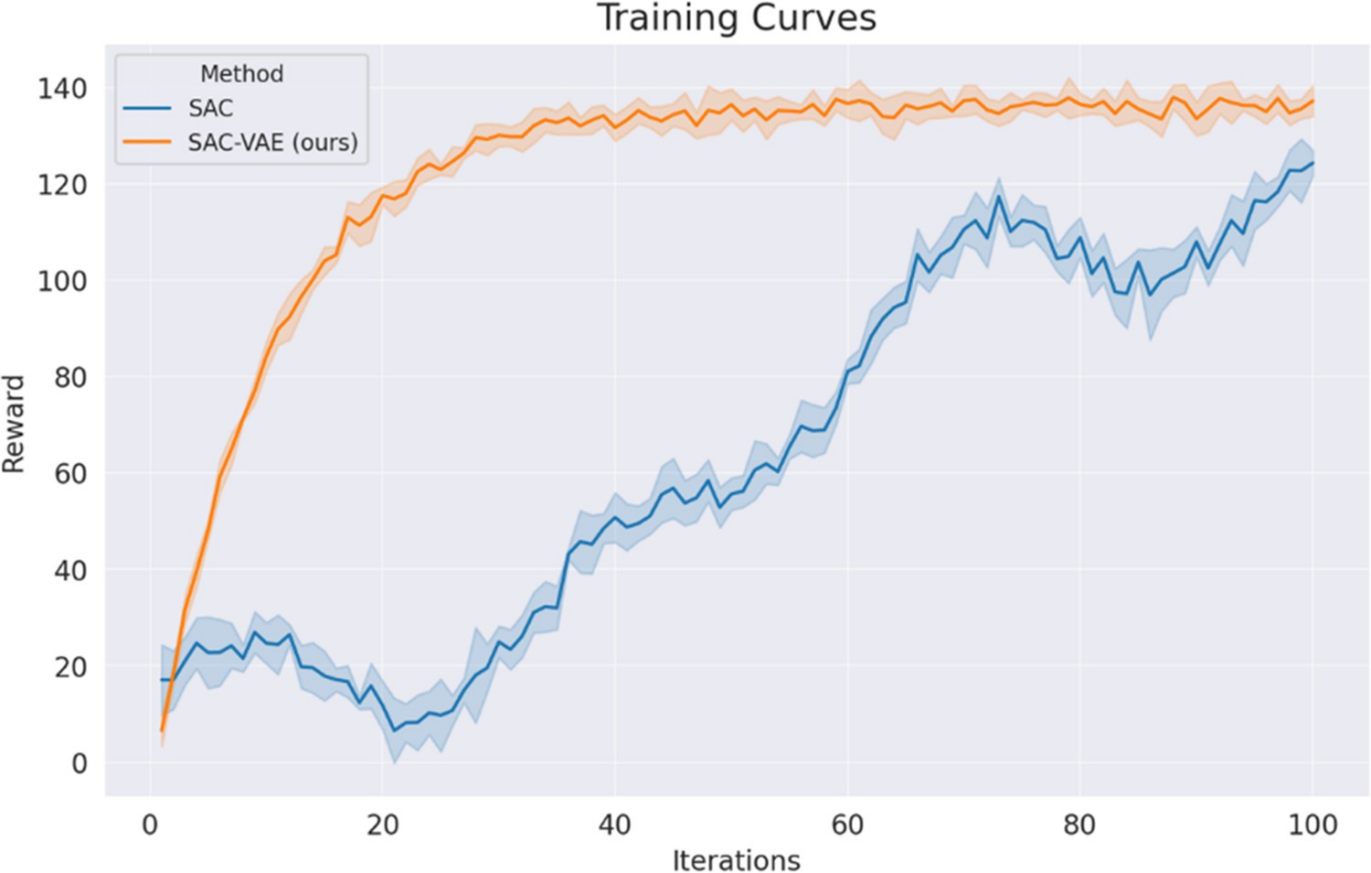
Training curves of comparison methods.

This section offers an experimental analysis focused on the training convergence speed and reward exploration capabilities of the SAC-VAE algorithm. To assess the algorithm’s stability post-VAE state reconstruction, the SAC serves as the baseline for comparison. Multiple tests were conducted using different random seeds, and the algorithm’s average learning performance was compared against the baseline across five distinct random seeds, as illustrated in Fig 3. The key parameters for both the SAC and VAE algorithms are delineated in Table 1.

**Table 1:** Main experimental parameters.

SAC	Number of neurons	Number of hidden layers	Discount factor	Learning rate	Soft update coefficient	Batch size	Entropy threshold	Optimizer
	256	3	0.99	0.03	0.005	128	0.9	Adam [19]
VAE	Number of neurons	Number of hidden layers	Optimizer					
	60	2	Adam [19]					

As indicated by the results presented in Table 2, the learning performance of the proposed SAC-VAE algorithm substantially outperforms that of the baseline algorithm. Specifically, with respect to the final reward metric, the SAC-VAE algorithm exhibits an improvement rate of 9.66% when compared to the SAC algorithm. Furthermore, in the context of training efficiency, the SAC-VAE algorithm reaches convergence in 116 minutes, thereby reducing the time to achieve a steady state by 59.86% relative to the baseline. Additionally, the SAC-VAE algorithm demonstrates enhanced training stability compared to the baseline SAC algorithm.

**Table 2:** Results of comparison methods.

	Final reward average value	Training time to reach convergence (minutes)
SAC	124.68	289
SAC-VAE	136.72	116
Improvement rate	9.66%	59.86%

Table 3 delineates the distribution of ROUGE-1, ROUGE-2, ROUGE-L and BLEU for each model under investigation. These results indicate that the SAC-VAE framework significantly outperforms the traditional SAC approach in summarizing legal documents. The integration of VAE allows for a more effective compression of the high-dimensional state space of legal texts, facilitating a more focused and efficient policy learning process. This is evident in the improved scores across ROUGE-1, ROUGE-2, and ROUGE-L metrics, which collectively suggest that SAC-VAE not only captures the essential content and details more accurately but also better preserves the structure and coherence of the original texts. The remarkable improvement in the BLEU score further underscores the SAC-VAE method’s ability to generate precise, relevant, and high-quality summaries. This metric, known for its emphasis on n-gram precision and the incorporation of a brevity penalty, indicates that SAC-VAE can effectively produce summaries that are both concise and closely aligned with the human-generated reference summaries.

**Table 3:** Performance of comparison methods.

Method	ROUGE-1	ROUGE-2	ROUGE-L	BLEU
SAC	0.587	0.462	0.496	0.285
SAC-VAE	0.833	0.716	0.607	0.683

### 5.3 The Impact of reconstruction with different compression scales

To evaluate the impact of the algorithm presented in this study on convergence speed and stability across varying degrees of feature compression, the SAC-VAE algorithm was tested at dimensionalities of 40, 50, 60, 70, and 80, respectively. For each dimensionality, three sets of trials were conducted using random seeds, and performance was assessed through the analysis of the results.

As evidenced by Table 4, the algorithm’s training efficiency is markedly enhanced at all levels of feature compression when compared to the baseline algorithm. Notably, at a compression scale of 60 dimensions, the algorithm achieves convergence in just 15 minutes. This represents the highest improvement rate in training efficiency, accelerating the time required to reach a steady state by 60.21%. Additionally, in terms of algorithmic performance, the rate of improvement in exploration capability exceeds 3% across all tested compression scales.

**Table 4:** Experimental results of SAC-VAE algorithm with different compression dimensions.

Compression dimensions	Training time to reach convergence (minutes)	Improvement rate compared with SAC	Final reward	Improvement rate compared with SAC
40	124	57.09%	128.73	3.25%
50	119	58.82%	130.65	4.79%
60	115	60.21%	135.86	8.97%
70	125	56.75%	139.93	12.23%
80	132	54.33%	132.36	6.16%1

### 5.4 Reconstructed state vector similarity analysis

In this section, we investigate the degree of similarity between the reconstructed, compressed state vectors and their original counterparts. We further elucidate the underlying reasons for the enhanced training performance observed with the reconstructed state vectors by examining the reconstruction distance metrics.

In the experiment, we analyzed the encoder networks of the VAE at compression scales of 40, 50, 60, 70, and 80 dimensions. The similarity between the original 2048 state samples and the corresponding output samples from the decoder network was quantified using Euclidean distance metrics. The results of this analysis are presented in Table 4.

Among the various compression scales examined, the encoder network with a 60-dimensional compression scale yielded the most favorable performance, exhibiting the highest mean similarity for the reconstructed states. As delineated in Table 5, the arithmetic mean of the similarity measure is 6.28. This finding corroborates the superior performance of the SAC-VAE algorithm when operating at a compression scale of 60.

**Table 5:** Reconstruction distance analysis.

Number of dimensions	40	50	60	70	80
Reconstruction error	6.98	7.12	6.28	6.85	6.72

## 6 Conclusion

This paper presents SAC-VAE, a groundbreaking framework designed to address the complexities inherent in legal summarization tasks. The architecture incorporates an unsupervised representation learning module that effectively reduces the original high-dimensional state space to a low-dimensional feature space, utilizing sample trajectories. Empirical evaluations reveal that the SAC-VAE framework, capitalizing on this learned low-dimensional representation, outperforms existing approaches in the domain of legal summarization. The marked improvements underscore the efficacy and innovation of the proposed SAC algorithm.

Furthermore, our work aligns closely with the United Nations Sustainable Development Goals, particularly Goal 16: Peace, Justice, and Strong Institutions. By enhancing the accessibility and comprehension of legal texts, SAC-VAE contributes to the democratization of legal information, facilitating broader public understanding and engagement with legal matters. This is crucial for fostering a more transparent, inclusive, and just legal system.

Looking ahead, we see opportunities to deepen collaboration with civil society and legal stakeholders to further refine our tool, ensuring its relevance and applicability in various legal contexts. Our work exemplifies how AI can be leveraged for societal good, bridging the gap between complex legal information and public accessibility. Future research could explore extending the SAC-VAE framework to multilingual legal documents and incorporating additional unsupervised learning techniques to further optimize feature space reduction. Moreover, real-time deployment of SAC-VAE in legal workflows would provide valuable insights into its impact on practical legal decision-making and document review processes.
